# Removing Alpha Case from Laser Powder Bed Fusion Components by Cavitation Abrasive Surface Finishing

**DOI:** 10.3390/ma18091977

**Published:** 2025-04-26

**Authors:** Rohin Petram, Conall Wisdom, Alex Montelione, Cole Nouwens, Dan Sanders, Mamidala Ramulu, Dwayne Arola

**Affiliations:** 1 Department of Materials Science and Engineering, University of Washington, Seattle, WA 98195-2120, USA; 2 Department of Mechanical Engineering, University of Washington, Seattle, WA 98195-2120, USA; 3 Sugino Machine Ltd., Toyama 936-8588, Japan

**Keywords:** additive manufacturing, alpha case, laser powder bed fusion, material removal, post-processing, surface treatment

## Abstract

Laser powder bed fusion (L-PBF) has become a highly viable method for manufacturing metal structural components for a variety of industries. Despite many attractive qualities, the rough surfaces of L-PBF components often necessitates post-processing treatments to improve the surface finish. Furthermore, heat treatments are generally necessary to control the microstructure and properties of L-PBF components, which can impart a detrimental surface oxide layer that requires removal. In this investigation, cavitation abrasive surface finishing (CASF) was adopted for the surface treatment of Ti6Al4V components produced by L-PBF and removal of the surface oxide layer. The surface texture, residual stress, and material removal were evaluated over a range of treatment conditions and as a function of the target surface orientation. Results showed that CASF reduced the average surface roughness from the as-built condition (R_a_ ≈ 15 µm) to below 5 µm as well as imparted a surface compressive residual stress of up to 600 MPa. The CASF treatment removed the alpha case from direct line-of-sight surfaces under a range of treatment intensity. However, deep valleys and surfaces at large oblique angles of incidence (≥60°) proved challenging to treat uniformly. Overall, results suggest that CASF could serve as a potent alternative to chemical treatments for post-processing of L-PBF components of titanium and other metals. Further investigation is recommended for improving the process effectiveness and to characterize the fatigue performance of the treated metal.

## 1. Introduction

Additive manufacturing (AM) through laser powder bed fusion (L-PBF) enables parts to be designed and fabricated rapidly, without costly tooling, which minimizes the time from part design to net-shape manufacturing [1,2,3,4]. The layer-by-layer build process of L-PBF supports nearly unlimited design complexity, a shortcoming of traditional forging and/or machining to achieve net shape, especially for titanium alloys [5]. Despite many attractive qualities of L-PBF, it struggles to produce metal that meets the mechanical properties of wrought components, especially in fatigue [6,7,8]. One of the primary reasons is the porosity in L-PBF [8,9]. In addition, the melting of powder layer by layer creates grain structures that exhibit anisotropic mechanical properties that depend on the part orientation relative to the build direction [10]. As such, a post-processing heat treatment is generally required.

Hot Isostatic Pressing (HIP) has become the predominant heat treatment for the post-processing of L-PBF components [11]. Through the superposition of elevated temperature at high pressure, HIP treatments can improve the grain structure and reduce porosity [12,13]. However, oxygen within the furnace environment can react with the parts and cause the formation of an oxidized layer on the surface. For titanium alloys this layer is often referred to as an “alpha case” due to the high concentration of alpha phase crystalline grains [14]. Due to the highly brittle nature of the alpha case, it can lead to the development of microcracks on the surface, which are detrimental to the fatigue properties of titanium alloys [15].

Another concern in L-PBF is that the process results in a layer of partially melted and sintered powder on the surface of parts. The surface texture can be influenced by the process parameters to some extent, but the high surface roughness is detrimental to the printed part quality [7,16,17,18]. A post-processing treatment capable of improving the surface quality is required for components that are considered stress-critical or will undergo cyclic loading.

Chemical milling currently serves as the standard in the aerospace industry for reducing the roughness and removing alpha case from Ti6Al4V components produced by L-PBF [19,20]. However, there are substantial environmental and health concerns in chemical milling due to the chemicals involved [21]. Additionally, while it is effective at removing alpha case [22], chemical milling does not introduce residual stress, a key to improving the fatigue resistance of metallic parts [23]. Shot peening is perhaps the principal surface treatment for introducing residual stress but requires line of sight, which is less likely for AM parts with complex geometry [24,25].

There are several other peening techniques used for surface treatments. For instance, water jet peening (WJP) [26,27] and abrasive waterjet peening (AWJP), a derivative of WJP [28,29,30], are potential alternatives. Cavitation peening is another technique that uses a cavitating jet of water to peen the surface through the implosion of vapor bubbles [31]. Cavitation processes involving laser pulses and ultrasonic waves have also been reported [31,32]. Although capable of introducing residual stress and improving the fatigue strength of metallic components, their propensity for reducing surface roughness is less clear; a potential limitation of all “shotless” type cavitation processes in application to metal additive components [33]. A comparison of the attributes of the most prominent surface treatments is presented in Table 1.

Cavitation abrasive surface finishing (CASF) is a form of cavitation peening whereby a high-velocity cavitating waterjet is introduced within an abrasive slurry tank. The dynamic jet embodies a cloud of cavitation bubbles, which energize the abrasive particles in the slurry to promote material removal and reduce the surface roughness. Implosion of the cavitation bubbles in contact with the target surface imposes localized plastic deformation and the introduction of compressive residual stresses in the near surface layers, which appear capable of improving the fatigue strength of additive components [34,35]. There are other potential advantages of CASF in application to L-PBF components. Since the treatment comes from the implosion of bubbles and not the jet itself, the treatment is less reliant on direct line of sight and can be directed into internal passageways. Furthermore, the CASF treatment utilizes water and abrasives, which pose no environmental concerns. Previous work on CASF [34,35] suggests that it may be possible to remove alpha case layers formed from heat treatment in addition to smoothing.

In this investigation, CASF treatments were performed on hexagonal bars of Ti6Al4V produced by L-PBF. Post-processing of the bars resulted in samples with and without an alpha case. The primary objective of this study was to evaluate the application of CASF for improving the overall surface quality, and removal of the alpha case layer specifically. The surface texture and surface integrity of the treated Ti6Al4V targets are presented with respect to the treatment feedrate, and the accessibility of the surfaces with respect to the incident jet. While various surface finishing processes have been previously investigated for the treatment of Ti-6Al-4V, two aspects of this investigation are novel. First, no prior work has been reported on the removal of titanium alpha case using CASF and the corresponding residual stress after treatment. Secondly, a novel approach was used to evaluate the effective stress concentration factor for the surface texture, which has not been applied to L-PBF parts before.

## 2. Materials and Methods

### 2.1. Materials

The hexagonal bars used in this study were produced with Grade 5 Ti6Al4V titanium powder and an EOS M290 printer (EOS, Krailing, Germany). The powder was used in prior production builds and thus was in the “reused” condition without provenance. Additional details regarding the vendor, number of prior builds or hours of exposure were not available. These details are not highly relevant to assess the efficacy of the CASF process. Nevertheless, the powder conformed to the requirements outlined in ASTM F2924 [36], which establishes the composition limits of potential contaminants. Eight hexagonal bars (hexbars) were produced from this powder for the investigation, all with width and length of roughly 2 cm × 10 cm, respectively. The bars were all printed vertically from the build plate.

Following printing, the hexbars were sectioned from the build plate and then subjected to a Hot Isostatic Pressing (HIP) cycle by a vendor within the USA (name withheld), at pressure and temperature of 190 MPa and 815 °C, respectively, and for two hours. Four of the hexagonal bars were exposed to oxygen during the HIP process, which resulted in the development of an oxide layer on the outer surface and regarded herein as an alpha case [11].

### 2.2. CASF Treatment

**Figure 1:**
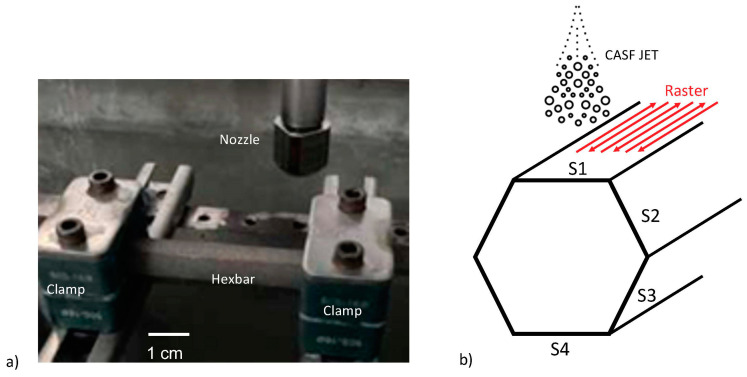
Details of the treatments performed. (**a**) experimental arrangement, (**b**) schematic of the Hexbar treatment path and surface labels relative to the jet. Surface S1 is considered the face as it is in the line of sight of the CASF jet.

The hexagonal bars were clamped in a simple supported arrangement (Figure 1) at each end and submerged in a slurry of water and alumina particles with between 30 and 40 volume % abrasive concentration. The abrasives consisted of aluminum oxide with a mesh number of # 200. The CASF treatment consisted of rastering the jet over one side of the sample six times with a spacing of roughly 4 mm between passes, as shown in Figure 1b. The initial and final passes extended outside the bounds of the bar to ensure treatment was consistent over the whole width of the face. The standoff distance from the nozzle to the S1 surface of the bar was 50 mm and the nozzle was rotated at 200 RPM. Treatments were conducted with jet traverse rates of 60, 70, 80, and 90 mm/min, one hexagonal bar at each rate. The CASF jet traversed along the major axis of the bars, which was perpendicular to the lay of the surface resulting from L-PBF.

### 2.3. Surface Texture

After completion of the CASF treatments, the surface roughness was evaluated using a commercial contact profilometer (Mahr MarSurf GD 25, Providence, RI, USA) with a MFW II Tip attachment having a 90° included angle and 2 µm radius of curvature. Three scans were conducted for each specimen. For each profile obtained, the average roughness (R_a_), ten-point roughness (R_z_), and peak to valley height (R_y_) were calculated according to ISO 4288 [37]. As defined by this standard, a cutoff length of 2.5 mm and traverse length of 15 mm were appropriate, permitted that the measured roughness was R_a_ ≤ 10 µm. Although some of the roughness measurements exceeded 10 µm, which calls for a cutoff length of 8 mm, the longer cutoff length was not feasible due to the required traverse length and limitation of the profilometer.

### 2.4. Stress Concentration Factor

The raw data from the contact profiles were used to identify prominent profile valleys. A graphical radius gauge was then used to estimate the radius of curvature at the valley root according to the methodology outlined in References [38,39]. For each line scan, the six most prominent valleys were selected as candidates for estimating the stress concentration over the evaluation length of 15 mm. A set of guidelines were followed to ensure an objective measurement of the concentration of surface stress. In general, one valley was selected every 2.5 mm, or for each cutoff length. In addition, the deepest valleys were selected within each cutoff length to result in the estimation of the largest stress concentration. Finally, each valley selected for measurement was at least 1 mm apart from neighbors to capture a larger distribution of the roughness scan.

**Figure 2:**
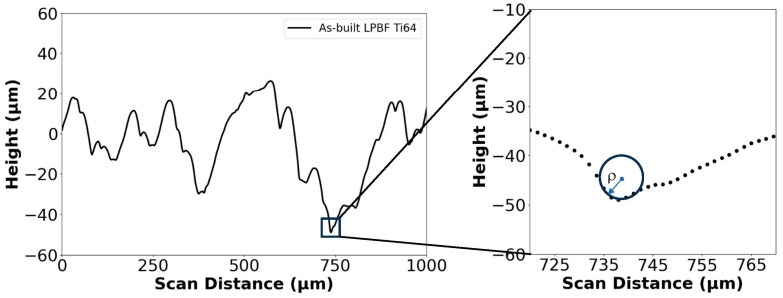
Identifying prominent valleys from surface profiles and measurement of the profile valley root radii (*ρ*) using a graphical radius gauge. The profile shown is from a hexbar in the as-built condition. Note that only 1 mm of the profile is shown to help view the geometry of individual valleys.

The root radius (*ρ*) of each valley identified for measurement was estimated as shown in Figure 2. A cursory examination of the uncertainty in valley root radius (*ρ*) estimation using this graphical approach showed that it did not exceed 10%. The effective stress concentration factor (${\bar{K}}_{t}$) was then estimated according to:

(1) $$\bar{K_{t}}=1+n(\frac{R_{z}}{\bar{\rho }})(\frac{R_{y}}{R_{z}})$$

where the *R_a_*, *R_z_*, and *R_y_* values refer to the arithmetic average, ten-point, and peak to valley roughness measurements, respectively, and the value ($\bar{\rho }$) refers to the average profile valley radius estimated from the six measurements. For both *R_a_* and *K_t_*, a one-way Analysis of Variance (ANOVA) was used to compare the measures and a value of *p* ≤ 0.05 was used to distinguish significance.

### 2.5. Material Removal

The material removal resulting from the CASF treatments was evaluated using line-scan surface profiles across regions of the surface that were masked to resist erosion. The abrupt change in surface height evident between the mask and treated region enabled the depth of material removed to be estimated along with the corresponding material removal rate.

### 2.6. Residual Stress Measurements

The residual stress in the treated surface of the Ti6Al4V samples was estimated using the sin^2^(*ψ*) method [40]. X-ray diffraction was performed using a Bruker D8 Discover X-ray Diffractometer (XRD) (Bruker, Billerica, MA, USA) with a copper target (Cu Kα) used for the X-ray source. The XRD generated X-rays at 50 kV and 1000 µA and focused the beam using a 0.5 mm collimator. A Pilatus 100 K large area 2D detector was used to capture diffracted photons from the [312] plane at 2θ = 110°. Each sample was measured at nine distinct angles with a full complement of *Φ* (0°, 45°, and 90°) and *ψ* (0°, 22.5°, and 45°) combinations. The scans were performed with a 2D detector capturing a 2θ range from 107° to 113°, which resulted in a total time elapsed per scan of 4 min [41]. The residual stress in the metal was estimated from the location of the diffraction peaks at these angles using Bruker’s software (Bruker, Diffrac.Leptos, V7.10.12, Billerica, MA, USA). The elastic constants used for the residual stress calculations including the Poisson’s ratio and bulk modulus were ν = 0.320 and E = 113 GPa, respectively.

### 2.7. Optical Microscopy

To characterize the extent of the alpha case and its removal, cross-sections of the treated hexbars were prepared for optical imaging. The cross-sections were mounted in black glass-filled epoxy (Allied 150-10105, Cerritos, CA, USA) in cylindrical molds. The exposed surfaces were then polished using silicon carbide abrasive mesh pads from #240 to #800 mesh (Allied 50 series, Cerritos, CA, USA). Thereafter, a final abrasive polish was performed using a 9 μm DiaLube diamond particle suspensions on a Struers MD-Dac pad (Struers, Cleveland, OH, USA), followed by a chemical attack polish on a Struers MD-Chem pad with a solution comprising 10 mL 0.05 μm colloidal silica, 0.5 mL 5% ammonium hydroxide, and a few drops of 40% hydrogen peroxide. After polishing, the samples were etched with Kroll’s reagent (etchant #192 of ASTM E407-7 [42]) for seven seconds to accentuate the grain boundaries and expose the alpha case, which appears white in contrast to the gray of the base metal. The duration of treatment to accentuate the grains and reveal case was established by preliminary testing. Optical images were obtained from these treated surfaces using an optical microscope (Olympus Model BX51M, Center Valley, PA, USA) at between 5Xand 50X magnification.

## 3. Results

### 3.1. Surface Texture

**Figure 3:**
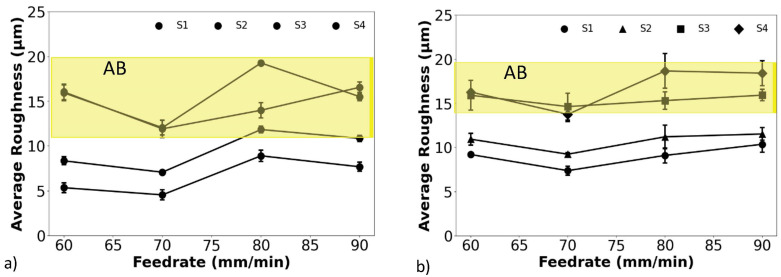
Average surface roughness (*R_a_*) after CASF treatment of the (**a**) control hexbars, and (**b**) hexbars with the alpha case. The yellow zone indicates the range in R_a_ of the as-built (AB) condition.

The average surface roughness of the hexbars after CASF treatment is shown in Figure 3a,b, respectively, for the samples without and with alpha case. The roughness is shown for the four primary surfaces, from S1 through S4 as shown in Figure 1b, and as a function of the treatment feedrates. For reference, the Ra values for the as-built (AB) condition are also shown in each graph as well. Apparent in this figure, the S1 and S2 surfaces exhibited the greatest reduction in roughness relative to the as-built condition, decreasing from nearly 15 µm to below 5 µm Ra for the surfaces without alpha case. The remaining two surfaces (S3 and S4) did not undergo appreciable reduction in roughness with CASF treatment, exhibiting an Ra consistent with the as-built condition. Apart from the generally lower roughness resulting from the slower feedrates at 60 and 70 mm/min there is no consistent trend in roughness with feedrate. Overall, there was a slightly greater reduction in roughness in the treatment of the targets without the alpha case.

**Figure 4:**
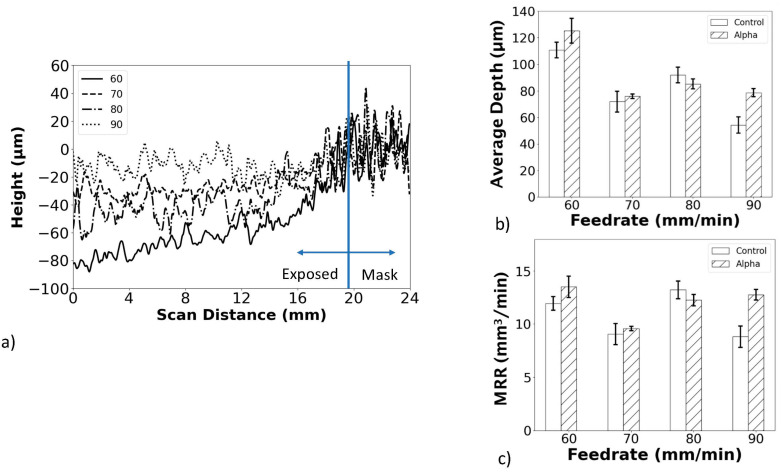
Estimating the material removal rate resulting from CASF. (**a**) Boundary of the treatment on the S1 surface, (**b**) average material removal depth, (**c**) effective material removal rate (MMR) on the S1 surface.

The transition from the treated area to the masked area of the targets is shown in Figure 4a. The depth of material removed from the CASF treatment is evident in this figure and the values for the S1 surface are reported in Figure 4b. The alpha case specimens exhibited greater material removal than the control specimens for most feedrates. The effective material removal rates (MRR) of the CASF process were estimated from the volume of material removed per treatment time and are shown in Figure 4c in terms of feedrate. Due to the larger depth of material removal for the alpha case specimens, the MRR is also generally greater for those specimens with case, with the exception of the treatments conducted at 80 mm/min.

**Figure 5:**
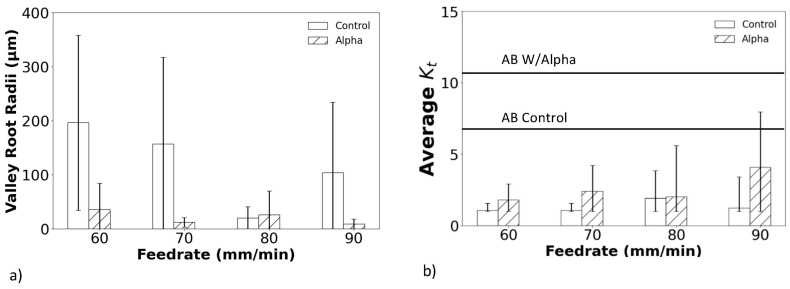
Changes in the valley characteristics with CASF treatment. (**a**) Valley root radii (**b**) average effective stress concentration factor. The horizontal lines indicate the average *K_t_* of the as-built (AB) condition.

Prominent valleys were identified in the profile height distributions obtained from surface S1 of the as-built and treated samples. These valleys were then used to measure the valley root radii (ρ) in support of estimating the ${\bar{K}}_{t}$, as shown in Figure 5a,b, respectively. In the as-built condition, the surfaces exhibited small valley root radii and comparatively high ${\bar{K}}_{t}$ values due to the surface characteristics resulting from the L-PBF process. After the CASF surface treatment, there was a significant increase in the valley root radii of the surfaces without alpha case, and with respect to the surfaces with alpha case. While improvements were achieved for the entire range of feedrates applied, the largest occurred at the lowest feedrate. As shown in Figure 5b, the increase in ρ resulted in substantial reductions in the ${\bar{K}}_{t}$ of the surface profiles regardless of whether the surfaces had alpha case or not. Nevertheless, a larger reduction in the ${\bar{K}}_{t}$ occurred in the targets without alpha case.

### 3.2. Alpha Case Removal

**Figure 6:**
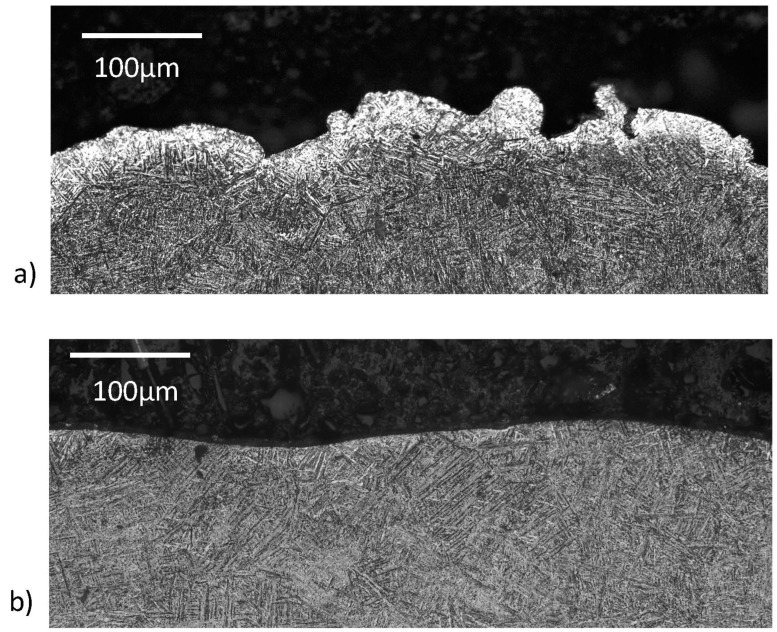
Alpha case depth revealed by cross-sectioning selected specimens and etching. (**a**) Micrograph of the hexbar surface S1 in the as-built (untreated) condition, and (**b**) the surface after treatment with CASF at 60 mm/min.

In addition to measuring the roughness and apparent stress concentration factors of the surfaces, the thickness of the alpha case layer was quantified before and after CASF treatment. A representation of the alpha case layers from cross-sectioning the treated specimens, etching and metallographic evaluation is shown in Figure 6a,b, respectively, for the as-built condition and after CASF treatment; this is the S1 surface of the hexbar treated with a feedrate of 60 mm/min. As is evident from the comparison of these two micrographs, the alpha case layer was completely removed. Meanwhile, at the faster feedrates of 80 and 90 mm/min, removal of alpha case appeared to be limited to approximately 50% of the surface, which was likely due to reduction in treatment intensity at the faster feedrates.

**Figure 7:**
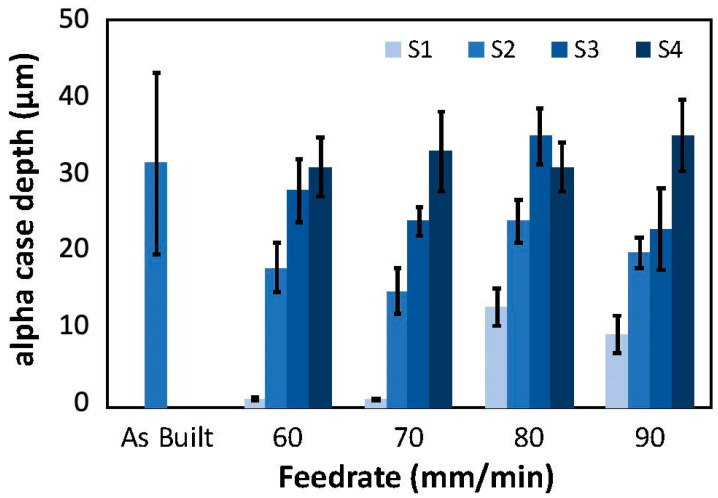
Thickness of the alpha case remaining after CASF treatment with respect to the feedrate. The surfaces S1 to S4 were shown in Figure 1. In the as-built condition, all surfaces have the same case depth.

Figure 7 shows results for alpha case thickness measurements post-treatment over the range in feedrates for all four primary surfaces, S1–S4. For lower feedrates of 60 and 70 mm/min, the alpha case was removed from approximately 90% of the S1 surface. There were small “pockets” of alpha case observed in some of the valleys of the treated surface. These pockets appeared to correlate with the deepest valleys, which were apparently shielded from the abrasives and material removal of the CASF process. As evident from the measurements for the surfaces S2 through S4 of Figure 7, the capacity for removing the alpha case decreased with a reduction in normal component of the surface perspective. The surfaces S3 and S4 exhibited limited evidence of alpha case removal with respect to the as-built condition.

**Figure 8:**
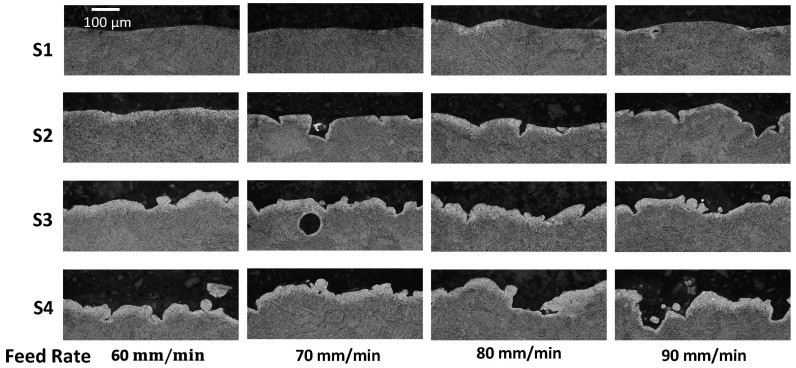
Micrographs of the hexbar surfaces S1 through S4 after CASF treatments and over the range in feedrates. These are cross-sections that have been etched to reveal the topography and the remaining alpha case.

A pictorial representation of the residual alpha case remaining on the treated surfaces of the hexbars over the range in feedrates is presented in Figure 8. These views are consistent with the perspective used to quantify the alpha case depth. In these photos, the alpha case removal is shown most clearly on the S1 face but is also apparent in the S2 face. It can be observed most clearly due to the substantial decrease in surface roughness on these faces. In contrast, the S3 and S4 faces appear to have limited removal of alpha case or improvement of surface roughness.

**Figure 9:**
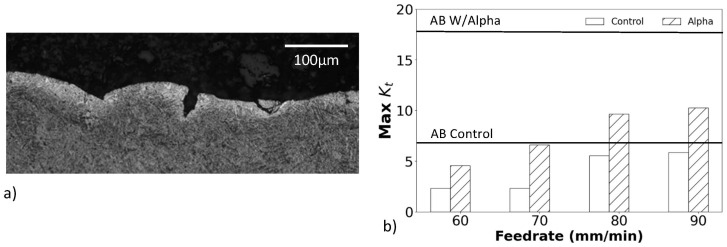
Residual features after CASF treatment. (**a**) Micrograph of CASF treated surface S1 with residual defects, (**b**) maximum *K_t_* of the specimens.

While the CASF treatment reduced the average roughness and apparent stress concentration for all surfaces, Figure 8 shows that residual valleys were evident on the surfaces with alpha case after treatment. Specifically, some of the sharpest valleys remained as shown in Figure 9a that could serve as crack nucleation sites. To quantify these residual features, the maximum *K_t_* was estimated using the profile valley radii measurements as shown in Figure 9b. These maximum *K_t_* values are substantially higher than the ${\bar{K}}_{t}$ values shown in Figure 5b for the treated surfaces and exceed those measured for the as-built condition.

### 3.3. Residual Stress

**Figure 10:**
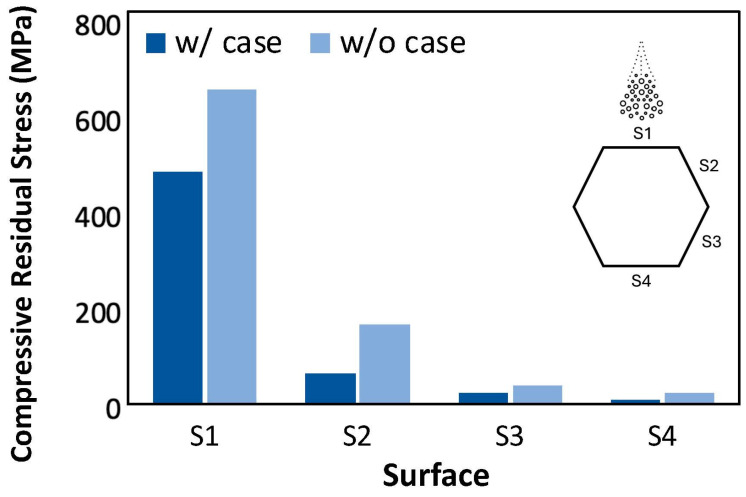
Comparison of the residual stress on the hexbar surfaces after CASF treatment for the bars with and without alpha case. All treatments represented in this plot were performed with a 60 mm/min feedrate.

Results of the residual stress measurements on the surfaces of the treated hexbar samples are presented in Figure 10. Both hexbars in this figure were treated by CASF with a feedrate of 60 mm/min. As is evident from Figure 10, the residual stress was consistently compressive in all the surfaces measured and it was greatest in the hexbars without alpha case. The residual stress within the S1 surface of the hexbar without the alpha case reached over 600 MPa, while that in the surfaces oriented obliquely to the incident jet was substantially lower after the CASF treatment. The compressive stress achieved within the S3 and S4 surfaces was less than 50 MPa, which was not significantly different from the residual stress of the as-built surface condition. The residual stress within the S3 and S4 surfaces would not be expected to influence the fatigue response apart from any benefit from the reduction in surface roughness.

**Figure 11:**
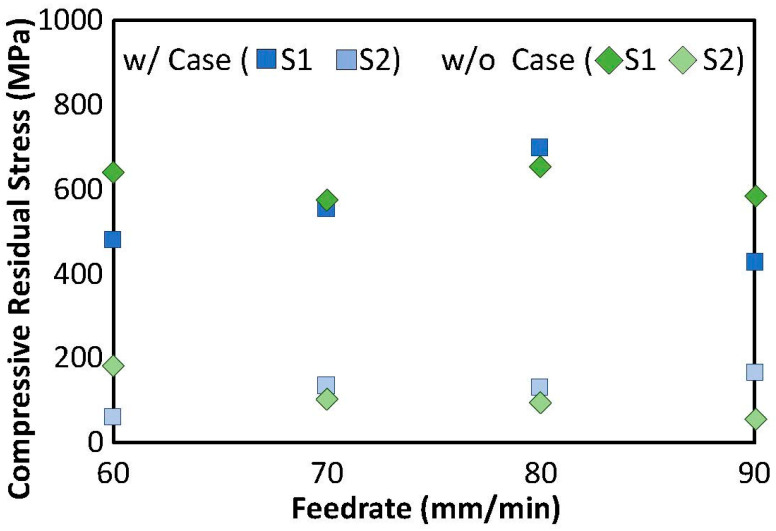
Residual stress on the S1 and S2 surfaces of the hexbars with and without alpha case over the range in feedrates. Note the greater importance of the surface orientation than the presence of alpha case.

Further evaluation of the residual stress distribution was performed to assess the influence of feedrate. Results from the residual stress measurements for the S1 and S2 surfaces are shown in Figure 11 as a function of feedrate for the hexbars with and without alpha case. There is no apparent trend in the residual stress with feedrate for both groups of hexbars. Interestingly, the evaluation of results over the range in feedrates shows that the residual stress was approximately equal in the targets with and without alpha case.

## 4. Discussion

The CASF treatment improved the overall surface quality of the Ti6Al4V components produced by L-PBF and removed most of the alpha case that resulted from a prior HIP treatment. However, there was some influence of the surface orientation relative to the incident jet and the treatment feedrate on the process effectiveness that warrant discussion.

Regarding changes in surface texture, the CASF process reduced the average roughness for both the control and alpha case samples over the entire range in feedrates explored (Figure 3). The influence of treatment on the variability in the average roughness is presented in Table S1 of the Supplemental Data. The reduction in *R_a_* was greatest on the S1 and S2 surfaces, which were both within the line of sight. These two surfaces were orthogonal and oriented obliquely with a 60° angle, respectively, to the jet. Hence, the target surface is not required to be orthogonal to the incident jet to improve the surface texture. In comparison, changes to the S3 and S4 surfaces were limited with respect to the as-built condition. That implies that while direct line-of-sight is not required, it is key to process efficiency and effectiveness. It is possible that improvements could be achieved if the jet’s energy is deflected onto surfaces that are not in the line of sight. That topic warrants further evaluation.

There was a reduction in Ra with a decrease in feedrate over the range in parameters examined. Indeed, the reduction in feedrate promoted higher treatment intensity (longer treatment time per unit area). A broader range in feedrates should be considered to pursue further reduction in the surface roughness beyond that achieved here. This effort should also explore the influence of the other process parameters including the abrasive size, standoff distance, and raster spacing, which are expected to contribute to the surface roughness as well.

There were limited changes to the Ra values of the S3 and S4 surfaces of the hexbars, independent of whether they had an alpha case layer. Hence, without further process modifications, the CASF process is not capable of achieving large improvements in surfaces that are completely obstructed from the direct line of jet impingement. It is important to treat surfaces within the line of sight or at least with impingement angle ≤ 60°, and within adequate proximity to the CASF cloud. Perhaps these limitations can be mitigated by including CASF treatment into the design process using the inherent flexibility and customization of AM design.

How does the improvement in Ra resulting from CASF compare to other treatments? With a reduction from approximately 15 µm to below 5 µm, results for the CASF process appear to be consistent with those possible from shot peening (3.36–4 µm) [23,24] and superior to those from laser shock peening (14–19 µm) [23,43]. Admittedly, the comparison of surface roughness resulting from different treatments is complicated by various factors, including the initial as-built surface roughness and the treatment parameters. Indeed, the results presented in Figure 4 suggest that further improvements in the CASF process could be achieved by a reduction in the feedrate. This could position CASF as a technology that rivals other prominent processes, including laser polishing and centrifugal finishing [23].

The profile valleys of the treated surfaces were characterized in terms of the dominant valley radii and the corresponding ${\bar{K}}_{t}$ they pose. Interestingly, while there was an increase in ρ and a reduction in ${\bar{K}}_{t}$ for all surfaces evaluated due to CASF, the hexbars without the alpha case exhibited a substantially larger increase in  ρ  relative to those with alpha case (Figure 5a). That suggests that an alpha case inhibits the treatment of the deepest valleys of the surface. Clearly, that is a consideration in components that will be subjected to cyclic loading and that the fatigue properties are of concern [44]. Treatment of components with alpha case for longer periods or utilizing a complementary approach that could improve the profile valleys specifically are potential options.

Optical microscopy confirmed that the majority (approximately 90%) of the alpha case was removed on the S1 surface that was treated with feedrates of 60 and 70 mm/min. At those lower feedrates the treatment intensity (duration of treatment per unit area) was large enough to achieve adequate material removal. However, at the faster feedrates of 80 and 90 mm/min, removal of alpha case appeared to be limited to approximately 50% of the surface, which was likely due to reduction in treatment intensity at the faster feedrates.

Small pockets of residual alpha case were identified, revealing incomplete removal even under the slowest conditions. That observation indicates that the oxide layer hinders treatment of deep valleys (Figure 8), even though the material removal rate was not influenced by the alpha case (Figure 4). A probable cause is the deep valleys of the as-built L-PBF surfaces shield some regions from the treatment cloud. Another possible cause is that the greater hardness of the alpha case requires direct contact of the abrasives for material removal. Deep valleys appear to restrict the abrasives from access and achieving material removal. It is conceivable that smaller abrasive particle sizes could have more success at penetrating deep valleys and removing the residual alpha case identified in the optical analysis. Alternatively, the treatment did not have enough time to fully remove the deepest valleys. Reducing the feedrate to increase the treatment time/intensity could remove the residual valleys. Regarding variation, the alpha case affected the CASF effectiveness and its ability to impose residual stress. While there is a possibility that the residual stress is lower in the deep valleys due to restricted access of the abrasives and cavitation bubbles, it is not possible to measure since the residual stress is an average over the spot size of the X-ray beam. The spatial consistency in residual stress in the CASF treated samples was measured on a single sample at multiple points along a 25 mm line. The coefficient of variation in residual stress was less than 0.10, which provided confidence that there was low spatial variation in residual stress overall on the CASF treated surfaces.

Results of the residual stress measurements suggest that there is an intricate relationship between the CASF treatment conditions, the alpha case, and the magnitude of residual stress. At the lowest federate, the samples with alpha case exhibited lower residual compressive stress after CASF treatment in comparison to the control samples. Apparently, the oxide layer inhibits the near-surface deformation that is necessary for imposing the residual stress. A two-stage CASF treatment may render improvements consisting of a parameter set that fully removes the alpha case, followed by a complimentary pass performed to maximize the surface residual stress.

With compressive residual stress exceeding 600 MPa recorded, CASF has shown that it can impart residual stress comparable to shot peening (348–650 MPa) [32,45] and exceeding that of tribofinishing (500 MPa). The compressive residual stress reported here for CASF is also slightly greater than that recently reported for laser cavitation peening (400–450 MPa) [32,46]. Therefore, CASF is a highly viable approach for the post-processing of L-PBF components of titanium [24,32,45,46] to achieve both reductions in roughness and introduction of compressive residual stress. These benefits would be expected for all metal targets processed by L-PBF, although with some dependence on the metal constitutive behavior.

There were two findings regarding the residual stress that warrant further discussion. Interestingly, there was no significant difference between the residual stress in the alpha case and control samples on the S1 and S2 surfaces. That appears to have occurred because most of the alpha case on these two surfaces was removed. The substantially lower residual stress on the S3 and S4 surfaces (Figure 10) shows that while CASF can treat surfaces that are not directly in the line of sight, the effectiveness is diminished. Either the abrasives are not delivered to the surfaces with the same momentum, or the cavitation bubbles are not brought in contact with the highly oblique surfaces to introduce appreciable surface deformation by implosion. Oblique surfaces that are shielded from the cavitation cloud cannot be treated as effectively, which was also observed in the measurements of surface roughness. Contrary to the minimal difference in surface roughness, the residual stress underwent a more substantial decrease in stress between the S1 and S2 faces. Clearly, the angle of impingement is key to the deformation invoked by implosion of the vapor bubbles on the surface and the residual stress that develops. With the current technology, if a component requires low roughness AND a high compressive residual stress, the surfaces should be oriented orthogonal to the incident jet to maximize the residual stress. If residual stress is a lesser requirement, then oblique treatment appears permissible.

Results show that a CASF treatment can improve the surface quality, remove alpha case, and simultaneously impose residual stress in metal components. Despite the value of these findings and evidence supporting the use of CASF for post processing components produced by L-PBF, there are limitations to the investigation. One potential concern is that this study was limited to the treatment of Ti6Al4V. The material removal and residual stress are undoubtedly dependent on the erosion resistance and mechanical properties of the substrate [47,48]. However, similar results are expected in the treatment of nearly all metals provided they have adequate ductility to undergo inelastic deformation under implosion of the cavitation bubbles. Regarding the surfaces resulting from L-PBF, all the surfaces treated in this investigation resulted from printing components vertically. Upskin and downskin surfaces are unique [18] and may require special consideration. While the surface residual stress for all conditions was compressive, the depth of residual stress was not quantified. That is an important quality that merits further exploration. Lastly, one of the primary purposes of conducting a post-processing treatment that imposes compressive residual stress is for improving the fatigue life. Fatigue testing is necessary to assess whether improvements in the fatigue behavior have been achieved by CASF treatment and to place them in perspective with regard to that reported for other comparable processes [49,50].

In summary, CASF can remove alpha case from the surface of L-PBF created components. Additionally, the combined benefits of improved surface roughness, reduction in the effective stress concentration posed by the topography and introduction of compressive residual stress suggests that it could enhance the fatigue resistance of metal additive components. Considering that it relies only on water and abrasives, avoiding the use of dangerous chemicals that pose risks to operators or the environment, CASF has substantial industrial potential. Nevertheless, before CASF becomes fully ready to implement for widespread adoption it will require parameter optimization to determine the ideal treatment conditions for the intended targets. That will be the focus of our future efforts.

## 5. Conclusions

An experimental investigation was performed to evaluate the changes in surface texture and residual stress of Ti6Al4V targets manufactured by L-PBF by CASF treatments and the potential for removing the alpha case layer. In summary:

i. The CASF treatment improved the surface texture of the treated hexbars as evidenced by a reduction in the average roughness and a decrease in the effective surface stress concentration posed by the topography. The treatment effectiveness was greatest for the surfaces oriented perpendicular to the incident jet, but some improvement was realized regardless of the surface orientation.
ii. The CASF treatment was successful at removing a majority of the alpha case layer from the targets. The greatest effectiveness was observed at low feedrate and for the surfaces treated with direct line of sight. Some residual alpha case was apparent on the hexbars after treatment that was located within the surface valleys, essentially shielded from the abrasive action. However, the alpha case did not significantly reduce the effective material removal rate of the CASF treatment.
iii. The CASF process introduced compressive residual stresses on the treated surfaces, reaching up to 600 MPa. Although the residual stress was highest in the hexbar targets without alpha case and when treated at feedrate of 60 mm/min, neither the alpha case or range of feedrate appeared to have a significant effect on the residual stress overall.
iv. The CASF process was most effective in treatment of the surfaces that were directly within the line of sight of the incident jet. There was also a reduction in surface roughness and introduction of compressive residual stress within the surfaces oriented obliquely to the jet (≤60°). However, there was limited change in the surfaces that were shielded from the line of sight.

## Figures and Tables

**Table 1:** Summary of the benefit and limitations of the various processes available for the surface treatment of components produced by L-PBF.

Surface Treatment	Surface Smoothing	Residual Stresses	Environment Impact	Line of Sight Required
Shot peening	Medium	Yes	None	Yes
Chem Milling	Large	No	Detrimental	No
Waterjet Peening	Medium	Yes	None	No
Cavitation Peening	Medium	Yes	None	Yes
CASF	Large	Yes	None	In Progress

## Data Availability

The raw data supporting the conclusions of this article will be made available by the authors on request.

## Supplementary Materials

The following supporting information can be downloaded at: https://www.mdpi.com/article/10.3390/ma18091977/s1. Table S1: Measures of variability in the average roughness of the CASF treated hexbars, including the Standard Deviation (Std Dev) and Coefficient of Variation (Coeff Var).
