# Supplementary material for: Removing Alpha Case from Laser Powder Bed Fusion Components by Cavitation Abrasive Surface Finishing
Source: Materials (Basel). 2025 Apr 26;18(9):1977. doi: 10.3390/ma18091977 (PMC12072853; doi:10.3390/ma18091977)
Supplement: Supplementary file 1 [file materials-18-01977-s001.zip › materials-3543370-supplementary.pdf]

materials

Supporting Information

Removing Alpha Case from Laser Powder Bed Fusion Components by Cavitation Abrasive Surface Finishing

Table S1. Measures of variability in the average roughness of the CASF treated hexbars, including the Standard Deviation (Std Dev) and Coefficient of Variation (Coeff Var).

**Control Specimens**	**Std Dev. (μm)**	**Coeff Var**
As-built	2.9	0.198
S1	0.55	0.104
S2	0.44	0.053
S3	0.88	0.054
S4	0.89	0.056
**Alpha-case Specimens**	**Std Dev. (μm)**	**Coeff Var**
As-built	2.1	0.126
S1	0.17	0.019
S2	0.68	0.062
S3	1.7	0.107
S4	0.21	0.013
